# Analysis of Sensory Properties in Foods: A Special Issue

**DOI:** 10.3390/foods8080291

**Published:** 2019-07-26

**Authors:** Edgar Chambers

**Affiliations:** Center for Sensory Analysis and Consumer Behavior, Kansas State University, 1310 Research Park Dr., Manhattan, KS 66502, USA; eciv@ksu.edu

**Keywords:** sensory, foods, consumer, descriptive

## Abstract

The sensory properties of foods are the most important reason people eat the foods they eat. What those properties are and how we best measure those properties are critical to understanding food and eating behavior. Appearance, flavor, texture, and even the sounds of food can impart a desire to eat or cause us to dismiss the food as unappetizing, stale, or even inappropriate from a cultural standpoint. This special issue focuses on how sensory properties, including consumer perceptions, are measured, the specific sensory properties of various foods, which properties might be most important in certain situations, and how consumers use sensory attributes and consumer information to make decisions about what they believe about food and what they will eat.

## 1. Introduction

Sensory analysis is an interdisciplinary science comprised of information and methods adapted from psychology, physiology, statistics, linguistics, food science, nutrition, medicine, chemistry, physics, sociology, anthropology, and a host of other fields. The antecedents of sensory testing go back many millennia, but modern testing of sensory properties of foods really began in earnest after World War I when the United States (US) military realized that soldiers came back from combat malnourished. This was caused, in part, because the food that was available to soldiers in military kitchens and through military rations had such poor sensory quality that the soldiers refused to eat it. In 1953 a symposium held in Chicago by the US Quartermaster Food and Container Institute of the armed forces was held to bring together various groups working to conduct sensory (including consumer) testing of foods [1]. In the proceedings of that conference, the organizers state “the impact of food testing methods has been felt across the nation. … the quality of food served both to the civilian population and to the Armed Services has been improved. Pretesting of new items and quality control testing of established products have already provided consumers with a more uniformly excellent food quality.” [1]

In the 1940s the US Army quartermaster corps scientists began studying human acceptance and how to measure it [2]. At the same time, scientists at Arthur D. Little, Inc. began promoting the use of descriptive sensory methods [3] for quantitatively measuring the sensory perception of food attributes. Cover [4] had already published work on a discrimination test, now called the paired comparison. In 1970 Mina McDaniel started work [5] on what could arguably be called the first dissertation in sensory sciences from within the food science field at the University of Massachusetts, Amherst. My own doctoral dissertation in 1980 and the resulting publication [6] was a short tome of just 36 pages with fewer than 20 references. There were few references because so little real science had been conducted at that point on sensory methods related to testing of actual foods. Heymann [7] recently published a “history” of sensory focusing primarily on the time since the 1940s and the many advancements made in sensory analysis since then. She comments on the work of various pioneers in the field. She mentions scientific organizations have taken hold, major conferences are being held, and scientists around the world are focusing on issues of product attributes, consumer acceptance and behavior, and ways in which to measure those aspects with more accurate and meaningful data. Yet, with the thousands of papers published in all those years, we still harken back to such fundamental papers as those describing the hedonic scale [2] and the flavor profile [8]. Fundamental information is still needed on many things.

Much of the sensory information we need can be divided into three categories, all of which are touched on in this group of papers. First, are studies that impact sensory methods. In science, methods are critical; without good methods we cannot collect good data. Four papers in this collection focus primarily on methods. Suwonsichon [9] treats us to a recent review of descriptive attributes. She updates older literature with papers in the past 5 years that focus on the description of various products including both human and pet foods. Such studies of attributes are essential for scientists to collect accurate, reproducible information on products across laboratories and countries. Of course, not all attributes happen simultaneously and Kuesten and Bi [10] provide us with ways to analyze data for multiattribute time intensity, particularly when used to compare to consumer acceptance in studies of the things that drive liking and disliking. For product developers, such information is critical in order to understand the attributes that must be present and at what levels to increase liking as well as to know which attributes should be reduced or eliminated. Torrico and others [11] provide us with information on carryover effects that can result in data that is less accurate because of sampling of prior products. Much has been written on the effects of this type of bias in all types of sensory studies, but this paper focuses on statistical ways to determine whether it may have occurred and for which attributes in consumer studies. Lastly, the temperature at which pain is associated with drinking coffee [12] provides both a practical method for assessing such perceptions as well as practical information on consumer liking of temperature when drinking coffee. The authors show that many consumers like coffee to be around 63 °C at consumption, which is only slightly less than the temperature (67 °C) that brings pain to many consumers. Furthermore, they tell us that, unfortunately, those temperatures are similar to the temperature at which hot foods/beverages can result in carcinogenicity.

The second area of focus is on the evaluation of products either by trained panels or consumers. This section is led by Yang and Lee’s review [13] of the evaluation of traditional and authentic foods. Those authors highlight a number of studies of products that are considered “ethnic” in nature, such as kimchi, artisanal cheeses, traditional sausages, and many more. Those types of products are key both within the countries and cultures they traditionally are consumed, but because food becomes more “global” and is introduced into new countries and cultures it must remain authentic, yet be appropriate for the new consumer. Wang and others focus on the effects of monosodium glutamate (MSG) in foods and the impact on flavor characteristics as well as liking [14]. Such information is helpful in understanding the nature of particular ingredients and their effect on both specific sensory properties as well as consumer acceptance. A further paper by Olsson et al. [15] shows the impact of processing on a specific food, in this case, the impact of emulsification intensity on sensory properties of mayonnaise. That paper is an example of the impact that processing can have on a product’s flavor and texture. The effect of water sustainability when growing almonds is evaluated by Lipan et al. [16]. This paper shows that water use can be reduced when growing almonds without an impact on the sensory properties or consumer liking of the nuts. The paper also transcends the description of the product and moves us into the next type of paper covered in this special issue, that of consumer behavior. The authors measured consumer acceptance of the concept of HydoSOStainable almonds and showed that consumers were potentially willing to pay more for the product. This is good news for growers and processors who could make more money with a product that, even though it is sensorially the same, has benefits that appeal to consumers outside the sensory aspects.

In addition to the research on sustainable almonds, another paper in this issue covers perception as it relates to behavior. The final paper in the issue focuses on “naturalness” of food ingredients [17]. Recent consumer trends have embraced the concepts of sustainability, organic production, and healthfulness. Although many people consider these aspects to make products “natural” or at least boost “naturalness”, the authors of this paper show clear evidence that consumers make assumptions about naturalness based on ingredients they may not understand. The authors point out that not understanding what an ingredient is, the use of “chemical-sounding names” and other issues impact whether consumers consider an ingredient natural, regardless of its actual source and processing.

These papers reference literally hundreds of other papers containing sensory data, which is an enormous leap from even 40 years ago. The world of sensory analysis continues to make headway into helping maintain a food supply that not only nourishes our bodies but also satisfies our minds and brings pleasure to our lives.
